# Development of a method for measuring water absorbency or release of food during mastication

**DOI:** 10.1186/s40064-015-1249-3

**Published:** 2015-08-28

**Authors:** Kazuyoshi Narita, Masahiro Hayashi, Hiroaki Masunaga

**Affiliations:** Tokyo Laboratory, EN Otsuka Pharmaceutical Co., Ltd., 1-11-10 Kinshi, Sumida-ku, Tokyo, 130-0013 Japan

**Keywords:** Mastication, Absorbency, Water release, Ease of swallowing, Mechanical measurement

## Abstract

Water release or absorption of food is related to ease of swallowing for individuals with difficulties in mastication or swallowing. The aim of this study was to establish methods to mechanically measure and predict water releasing or absorptive tendency during mastication. There were ten ingredients used. Six, Japanese radish, carrot, potato, salmon, chicken, and scallops were typically heated. The remaining four, apple, bread, cookies and kamaboko were used as is. Eight grams of water was added to 8 g of the ingredient, which was blended for 1 s in a mixer. After blending, the mixture was centrifuged or compressed using a texture analyzer machine. Ingredients were weighed before and after processing without water, and the percent increase in weight was calculated using the weight of the ingredients. Results demonstrated that three ingredients (Japanese radish, carrot, apple), which have strong tendencies for releasing, showed lower percent increases in weight, while two ingredients (cookies, bread), which have strong tendencies for water absorption, showed higher percent weight increases. The other five ingredients (potato, kamaboko, salmon, chicken, and scallops), which have no water releasing or absorption tendencies, showed mid-value percent increases in weight. The tendencies using all treatment methods were the same as during mastication. The percent increase in weight using two processing methods strongly correlated with increased rates of mastication, and demonstrated uncertainty equal to that of mastication. These methods may be helpful in establishing an index for ease of swallowing for classified diets in patients with dysphagia.

## Background

When food is masticated and mixed with saliva, salivary fluid is absorbed into the food, whereas moisture contained in the food is released into the mouth as the physical structure of the food is altered. Mastication make the food bolus. Residual liquid in the mouth subsequently facilitates the swallowing of food. The residual liquid, however, is also known to cause aspiration or asphyxia, depending on the amount released in elderly people with impaired masticatory and swallowing function and in infants for whom this function is still immature (Feinberg et al. 1996; Joan 2006). In rheological terms, bolus hardness rapidly decreased as the masticatory sequence progressed, by contrast, adhesiveness and cohesiveness regularly increased until the time of swallowing (Peyron et al. 2011). Foods with much water release are divided into bolus and water in mastication, and foods with much absorption of saliva in mastication make bolus of high adhesiveness, both are not good for elderly people or infants. There was a report that water content of cereal boluses collected just before swallowing was likely to depend on the person who ate the cereals and not depend on the type of the cereals eaten (Loret et al. 2011). Therefore, determining the level of water release or absorption in chewing food and predicting the difficulty in swallowing before each meal is clinically important. The hypothesis of this study was existence of methods of mechanically measurement for predicting water releasing or absorptive tendency during mastication. Conventional methods of determining the level of syneresis, water release, etc. of foods, including determining the surface and content of food include: (1) carefully placing food on filter paper or wiping food on filter paper (Umene et al. 2015), and (2) compressing food using a creep meter (Nohara et al. 2010). Another method of determining the water absorption of food is to immerse polished rice in water (Miwa et al. 2002). By using these conventional methods, however, measuring or predicting levels of water release from food and absorption of saliva into food in the mouth during food intake at the same time is not feasible. During mastication, movements of the tongue also contribute to bolus formation (Koshino et al. 1997), and maximum tongue pressure is reported to be approximately 40 kPa in normal individuals (Hayashi et al. 2002). Since conventional methods do not consider the influence of the tongue, properly predicting and understanding this influence on mastication and swallowing during food intake is difficult. For these reasons, we devised a new method to appropriately assess behaviors of water in the mouth during mastication and swallowing, including water release and absorption of foods, by classifying the characteristics of foods into the following three groups: (1) high water release, (2) intermediate, and (3) high water absorption, assuming (1) high water release and (3) high absorption foods are not suitable for people with dysphagia, while (2) intermediate foods are suitable.

## Results

### Measurement of salivary secretion during mastication

Of the 10 ingredients prepared, bread, the most absorptive sample, was chewed to determine the percent weight after mastication (Table 1).

**Table 1:** Weight and weight ratios after mastication of bread, n = 20

Weight ratio w/w%	Weight of bread			
	2.5 g	5 g	7.5 g	10 g
Average	172.1	155.6	141.7	131.4
SD	15.6	6.0	7.2	3.7
+2σ	203.3	167.7	156.2	138.9
+3σ	218.9	173.7	163.4	142.6

Results showed that the percent weight (mean + 3σ) was more than 200 % when the sample size was 2.5 g and less than 200 % when the sample size was 5, 7.5, and 10 g, indicating that percent weight after mastication increased up to 200 % when the weight of food was 8 g.

### Particle size measurement

**Fig. 1:**
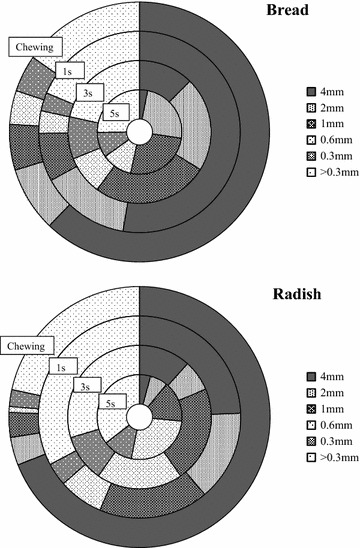
Particle size distributions after blending in a mixer, n = 3

**Fig. 2:**
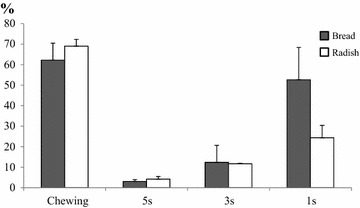
Weight percentage of 4 mm particle sizes after blending in a mixer, n = 3

Figures 1 and 2 show the size distributions of simulated bolus particles determined by the sieve method. Size distributions of particles when bread was blended in a mixer for 1, 3, or 5 s are expressed in dry weight. The size distribution of bread blended for 1 s, which was most closely correlated with mastication, was considered to be most similar to that of chewed bread. The size distributions of Japanese radish showed a similar trend.

### Measurement of water absorption and release

**Fig. 3:**
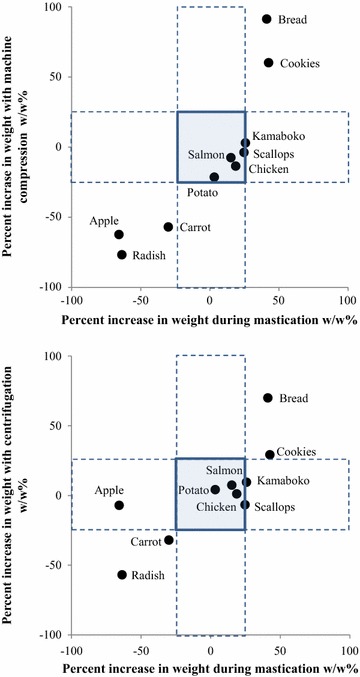
Scatter plot of percent increases in weight. The scatter plot represents percent increases in weight during mastication on the horizontal axis and percent increases in weight with machine compression or centrifugation on the vertical axis. *Squares* in the figure show the range of both values between −25 and +25. *Rectangles with dashed lines* show the range of one value between −25 and +25

To determine whether the samples exhibit water absorption or release, increases in the weight of each sample were calculated (Table 2; Fig. 3). A comparison of weights before and after treatment showed that ingredients that exhibited water release (high water release rate: Japanese radish, carrot, and apple) showed lower percent increases in weight (<−25 %), while those that exhibited water absorption (high absorption rate: cookies and bread) showed higher percent increases in weight (≥25 %), and those that exhibited neither of these properties (intermediate: potato, kamaboko, salmon, chicken, and scallops) showed intermediate values (−25 to 25 %). These tendencies did not vary among treatment modalities, with the exception of the centrifuged apple which showed a relatively large increase in weight. The percent increase in the weight of each ingredient varied depending on the method of treatment. When the ingredients are divided into high water release, intermediate, and high absorption groups, the order of samples within each group determined based on the magnitude of the percent increase in weight may be reversed depending on the treatment method used (for example, in the intermediate group, the increase in weight of chewed chicken was greater than that of chewed salmon, and the order is reversed (salmon > chicken) when these ingredients are treated using other methods). The order of samples from different groups determined based on the magnitude of the percent increase in weight; however, it was not reversed regardless of which treatment method was used, including mastication. Statistical comparisons of the 10 ingredients in terms of percent increase in weight from baseline revealed that all treatment methods were closely correlated with mastication (correlation coefficient of >0.7). Although the level of uncertainty of each treatment method calculated by the mean-value method (JCSS Guide to the Estimate of Uncertainty Hardness / Rockwell Hardness Appendix 1.4 2013) was lower than that of mastication, there were no significant differences.

**Table 2:** Percent increases in weight of 10 ingredients, w/w%, mean ± SD, n = 5

Samples	Mastication	After blending	
		Compression by machine	Centrifugation
Apple	−65.8 ± 1.0	−62.4 ± 0.9	−7.1 ± 5.5
Radish	−63.7 ± 5.0	−76.8 ± 4.2	−56.9 ± 4.7
Carrot	−30.1 ± 5.4	−56.9 ± 3.3	−32.0 ± 4.7
Potato	3.2 ± 3.7	−21.4 ± 5.5	4.2 ± 5.7
Salmon	15.2 ± 5.3	−7.5 ± 12.9	7.4 ± 8.2
Chicken	18.7 ± 13.0	−13.5 ± 7.1	1.1 ± 3.4
Scallops	24.7 ± 9.8	−3.8 ± 5.0	−6.7 ± 2.1
Kamaboko	25.7 ± 3.9	3.0 ± 3.1	9.5 ± 1.3
Bread	41.1 ± 7.8	91.3 ± 8.5	70.0 ± 9.5
Cookies	42.5 ± 7.1	60.2 ± 4.9	29.2 ± 3.5
Average deviation of 10 ingredients	6.3	5.8	4.9
Correlation coefficient r between mastication and the treatment	–	*0.8726	*0.7659

* Significant (p < 0.01) positive correlation between mastication and the treatment

## Discussion

Each sample was placed in a mill mixer, blended with water that had the same weight as the sample, and then compressed or centrifuged, which allowed the estimation of the level of and tendency toward water absorption and release in the mouth during food intake. The rate of increase in weight was used as an indicator of tendency for water absorption or release. When the ingredients tested were divided into the high water release, intermediate and high absorption groups, the rate of increase in weight was lowest in the high water release group, followed by the intermediate group, and highest in the high absorption group, regardless of whether the samples were chewed, mechanically compressed, or centrifuged. Ingredients in the high water release group can cause choking and aspiration due to water release in the mouth, while those in the high absorption group are difficult to swallow and will likely remain in the mouth due to absorption of saliva, and those in the intermediate group can be assessed as relatively adequate for swallowing with adequate water release. The method for measuring the rate of increase in weight used in the present study is considered to allow for proper evaluation of ease of swallowing, in particular water behavior in the mouth, including release of water from food and absorption of water into food during mastication and swallowing, by classifying food into high water release, intermediate, and high absorption groups based on their characteristics.

Comparisons of the rate of increase in weight of each ingredient after mastication and weight after each of the other treatment methods show that four ingredients tested in the machine compression group and one ingredient tested in the centrifugation group yield corresponding inverse values (positive versus negative) when tested in the mastication group, suggesting that, under the measurement conditions in this study, centrifugation allowed for more accurate water release and absorption measurement than mechanical compression. The rate of increase in the weight of ingredients that fell into the intermediate group was within the range from −25 to 25 %, regardless of whether these ingredients were chewed, mechanically compressed, or centrifuged, suggesting that the rate of increase in the weight of food that exhibits water release or absorption adequate for swallowing would be within this range.

### Measurement of salivary secretion during mastication

In view of the weight of bread measured after mastication and a report stating that salivary secretion was approximately up to 8 mL/min in adults (Gavião et al. 2004), estimating that the appropriate amount of water required to be added to 8 g of each sample in the present study was 100 % of the weight of the sample (i.e., 8 g) was considered reasonable.

### Particle size measurement

Particle size distributions of bread and Japanese radish after blending show that the correlation coefficient for Japanese radish was lower than that of bread treated using the same method, suggesting that Japanese radish was blended more finely than bread using the same treatment method, as in mastication, due to the softness of this food compared to bread (the tensile strength of bread is approximately 800 kPa, Nakano and Oba 1995, and that of cooked Japanese radish is approximately 600 kPa, Toyama et al. 1999). The possibility exists that the blending force needs to be changed according to the type food to be tested.

### Measurement of water absorption and release

The results of the preliminary study on the rate of increase in weight show that the greater the compression pressure, the higher the overall rate of increase in weight. For mechanical compression (490 N, approximately 270 kPa) and centrifugation (10,000×*g*, 250–400 kPa), the pressure was set to about 6–10 times normal tongue pressure (approximately up to 40 kPa, Hayashi et al. 2002). The inverse differences in the corresponding rates of increases in weight in the mastication group show that centrifugal acceleration was adequate in the centrifugation group, in which the differences were small, while the compression pressure was too high in the mechanical compression group, in which differences were more significant.

In the measurement of rate of increase in weight, only the centrifuged apple showed a relatively large increase in weight, probably due to the specific gravity of apple, which was lower than that of water (0.641, Examples of Density 2015), and due to bolus particles that should have originally been precipitated by centrifugation, which remained suspended and not separated in the supernatant. The percent increase in weight of the five ingredients which exhibited “intermediate” water release or absorption was slightly positive, likely due to the saliva that clung to food during mastication, thereby increasing the weight of food (Richardson and Feldman 1986) due to the higher viscosity (5–80 mPa s, Simon et al. 2011) relative to water (0.2–2 mPa s, Joseph et al. 1978).

The percent increase in food weight was closely correlated with mastication, regardless of the treatment method, suggesting that water behavior in the mouth during actual mastication (Woda et al. 2006) was well simulated by a series of food processing operations, from the addition of water, blending, to compression, allowing for absorption of water into food that was able to absorb water and allowing water separation from foods that exhibited water release caused by pressure. The levels of uncertainty of measurements using the treatment methods studied were lower than those for mastication, suggesting the possibility that water behavior in the mouth may be reproduced, since measurements using these treatment methods are as variable as or less variable than measurements of mastication. When food was grouped according to levels of water release and absorption, all of the ingredients tested were successfully classified into three groups (high water release, intermediate, and high absorption), regardless of the treatment method, and there were no differences in group composition among the treatment methods. Thus, it is evident that measuring the rate of increase in food weight is extremely useful in determining and predicting whether water is separated from foods or whether foods absorb saliva in the mouth.

## Conclusions

In order to properly evaluate the influence of water behavior during food intake on mastication and swallowing, we evaluated measurement methods to predict the level of water absorption into foods or release from foods during mastication. The study concluded that the method of determining pre-treatment and post-treatment weights and calculating the rate of increase in food weight closely correlated with mastication and results obtained using this method were as accurate as those by mastication. By using this method, food was treated as follows: each ingredient was placed in a mixer, blended with water that had the same weight as the material, and mechanically compressed or centrifuged. This method is considered to be effective in predicting whether food exhibits water release or absorption of saliva, etc. in the mouth during mastication.

## Methods

### Samples

Among commercially available food materials used as ingredient test samples, kamaboko, cookies, bread, and apple (Fuji) were used as is, and Japanese radish, carrot, potato (May Queen), salmon (silver salmon), chicken (breast meat), and scallops (adductor muscle) were cooked using commonly used methods. Table 3 shows nutrition facts of 10 ingredients.

**Table 3:** Nutrition facts of 10 ingredients, w/w%

Samples	Water	Protein	Fat	Carbohydrate
Apple	84.9	0.2	0.1	14.6
Radish	94.8	0.5	0.1	4.0
Carrot	89.1	0.6	0.1	9.6
Potato	81.0	1.5	0.1	16.8
Salmon	56.7	25.2	15.8	0.4
Chicken	70.0	25.0	4.1	0.0
Scallops	76.8	17.6	1.9	1.9
Kamaboko	74.4	12.0	0.9	9.7
Bread	38.0	7.9	4.2	49.0
Cookies	2.5	6.2	27.0	64.0

Cookies were based on data of Morinaga & Co., Ltd. (Moonlight Biscuit Morinaga & Co., Ltd. 2015), Bread were based on data of Shikishima Baking Co., Ltd. (Products lineup of Chojuku bread 2015), and other 8 ingredients were based on Standard Tables of Food Composition in Japan Fifth Revised and Enlarged Edition (Ministry of Education, Culture, Sports, Science and Technology 2008)

Japanese radish (high water release): A raw Japanese radish was cut into 15 mm cubes and cooked in boiling water (100 °C) for 1 h.

Carrot (high water release): A raw carrot was cut into 15 mm cubes and cooked in boiling water (100 °C) for 1 h.

Potato (May Queen) (low water release or absorption): A raw potato was cut into 15 mm cubes and cooked in boiling water (100 °C) for 15 min.

Kamaboko (low water release or absorption): Commercial kamaboko (Himekama (white), Kibun Foods Inc.) was cut into 5 mm thick slices.

Cookies (high water absorption): Commercial cookies (Moonlight, Morinaga & Co., Ltd.) were used as is.

Bread (high water absorption): Commercial bread (Chojuku 6 slices, Shikishima Baking Co., Ltd.) was cut into 15 mm cubes, each with a crust on one side.

Apple (Fuji) (high water release): A raw apple was cored and cut into 8–16 wedges.

Salmon (silver salmon) (low water release or absorption): Commercially available silver salmon slices were grilled with salt in an oven at 190 °C for 8 min.

Chicken (breast meat) (low water release or absorption): Commercially available chicken breast meat was steamed like steaming with wine at 100 °C for 5 min.

Scallops (adductor muscle) (low water release or absorption): Commercially available scallop adductor muscles were steamed like steaming with wine at 100 °C for 5 min.

### Subjects

The subject was 32 years old, good general and oral health after an information session, where he signed a consent form. He was free of any dental pathology, masticatory disorder and displayed a normal occlusion. The subject was asked to put the whole sample in his mouth at once. The subject was asked not to eat during at least 60 min before the experiment in order that hunger or the digestion stages do not influence his mastication behavior.

### Measurement of salivary secretion during mastication

To determine the appropriate amount of water required to cause water absorption into food, the amount of saliva absorbed was measured using bread, the most absorptive sample among those prepared.

In a preliminary study, the number of mastication cycles needed to prepare 3 g of bread for swallowing was 40 on average, which corresponded with a report that stated the mean number of mastication cycles needed to prepare 3 g of bread for swallowing was 37.6 ± 9.9 (Gavião et al. 2004). Based on these results, pieces of bread amounting to a mouthful (2.5, 5, 7.5, and 10 g) were prepared. After measuring the pre-treatment weight, each bread sample was placed in the mouth, chewed 100 times, and then spat out; that is, the number of mastication cycles was much greater than the typical number needed to prepare 3 g of bread for swallowing (40 cycles on average). The weight of the chewed and spat out sample (post-treatment weight) was measured. The percent weight after mastication was calculated using the formula shown below:

$${\text{Weight after mastication }}\left( {{\text{w}}/{\text{w}}} \right) \, \% \, = {\text{ Post-treatment weight }}\left( {\text{g}} \right)/{\text{ Pre-treatment weight }}\left( {\text{g}} \right) \, \times \, 100$$

Weight after mastication (w/w) % became a base of quantity of tap water for blending.

### Preparation of the bolus

The weight of a mouthful of each sample was set at 8 g based on the size of bread, the sample with the largest volume per weight among the 10 ingredients studied, which corresponded with a report that the weight of a mouthful of bread was 6.43 ± 2.19 g in adults (Yagi et al. 2006).

1. Mastication: In the preliminary study, the number of mastication cycles needed to start swallowing varied among the samples, from 30 to 45 cycles. To prepare post-treatment samples, 8 g of each sample was placed in the mouth, chewed as many times as required to prepare the materials for swallowing, and then spat out. Saliva and liquid produced during mastication were separated from samples as much as possible.
2. Blending: According to the result of measurement of salivary secretion during mastication, 8 g of each sample was blended with 8 g of tap water using a mill mixer (IFM-800DG with a micron container, Iwatani Corporation).

### Particle size measurement

The size distributions of bolus particles of chewed and blended samples were analyzed using a standard sieve method (Tate et al. 1994). Specifically, simulated bolus samples were sieved under running water using sieves with 5 different size openings (4.00, 2.00, 1.00, 0.60, and 0.30 mm, SANPO), dried in a steam/convection oven (RATIONAL) at 70 °C (dry mode) for 2 h, and then weighed using an electronic scale. Based on the weights of the samples, the size distributions of simulated bolus particles were analyzed.

### Compression

1. Mechanical compression: Three layers of the blended samples were wrapped in sterile gauze, and fixed on a piece of filter paper (ADVANTEC FILTER PAPER No. 2 φ110/Toyo Roshi Kaisha, Ltd.) set on a texture analyzer (SMS) equipped with a round-bottom plunger (φ = 48 mm). The samples were then mechanically compressed by applying a 490 N load, which was determined based on results from the preliminary study, from above for 10 s. As with the samples, sterile gauze moistened with water was also compressed and used as a blank sample. The post-treatment weight was obtained by weighing the compressed samples with sterile gauze, and subtracting the blank from the weight. The 490 N load, as used here, is equivalent to 270 kPa, which is approximately seven times normal tongue pressure (up to 41 kPa). Unlike tongue, surfaces of plungers and bases are flat, and there is little possibility of stress concentration and there is weaker strength degradation of bolus in mechanical compression.
2. Centrifugation: Blended samples were placed in centrifuge tubes, and centrifuged for 10 min at 10,000×*g*; the acceleration speed was determined based on results from the preliminary study. Water supernatant was removed, and the samples were weighed to determine post-treatment weights. The acceleration of 10,000×*g* is equivalent to 250–400 kPa, which is approximately 6–10 times normal tongue pressure (up to 41 kPa). Unlike tongue, surfaces of tubes are smooth and curved, and there is little possibility of stress concentration and there is weaker strength degradation of bolus in centrifugation.

### Measurement of water absorption and release

The rate of increase in weight was calculated for each of the treated samples using the formula shown below. Lower percent increases in weight suggest water release and higher percent increases in weight suggest water absorption. Table 4 shows an example of the calculation.

$${\text{Rate}}\;{\text{of}}\;{\text{increase}}\;{\text{in}}\;{\text{weight}}\;\left( {{\text{w}}/{\text{w}}\% } \right) = \left( {{\text{post-treatment}}\;{\text{weight}} - {\text{pretreatment}}\;{\text{weight}}} \right)/{\text{pre-treatment}}\;{\text{weight}}\; \times \; 100$$

**Table 4:** Example of calculation of percent increase in weight

Samples	Weight before treatment (g)	After treatment		Percent increase in weight (%)	Water release or absorption
		Weight (g)	Increase in weight (g)		
Apple mastication 1	7.79	2.68	−5.11	−65.6	Water release
Apple mastication 2	7.99	2.73	−5.26	−65.8	Water release
Cookies mastication 1	8.23	11.00	2.77	33.7	Absorption
Cookies mastication 2	8.37	11.76	3.39	40.5	Absorption

“Percent increase in Weight” is a calculated value for classifying ingredients into water release or absorption tendency during mastication

### Statistical analysis of percent increases in weight of 10 ingredients between mastication and compression by machine and between mastication and centrifugation

For the correlation coefficient analysis, a non-correlation test of Pearson’s product-moment correlation coefficient was used. For a comparison of differences in standard deviation between treatment methods, uncertainty regarding indirect verification was estimated using the mean-value method (JCSS Guide to the Estimate of Uncertainty Hardness/Rockwell Hardness Appendix 1.4 2013). Specifically, the uncertainty of (measurement of each ingredient—mean value of each ingredient) for all of the ingredients was calculated using the mean-value method, and an F-test was used to determine whether there was a significant difference. The level of significance was set at p < 0.05.
